# Modified Membranes for Redox Flow Batteries—A Review

**DOI:** 10.3390/membranes13090777

**Published:** 2023-09-01

**Authors:** Misgina Tilahun Tsehaye, Ramato Ashu Tufa, Roviel Berhane, Francesco Deboli, Kibrom Alebel Gebru, Svetlozar Velizarov

**Affiliations:** 1 Separation and Conversion Technology, Flemish Institute for Technological Research (VITO), Boeretang 200, 2400 Mol, Belgium; 2 Department of Environmental Engineering, University of Calabria (DIAm-UNICAL), Via P. Bucci CUBO 44/A, 87036 Rende, Italy; 3 Department of Chemical Engineering, KU Leuven, Celestijnenlaan 200F, 3001 Leuven, Belgium; 4 Lehrstuhl für Technische Chemie II, University of Duisburg-Essen, 45141 Essen, Germany; 5 LAQV-REQUIMTE, Chemistry Department, NOVA School of Science and Technology (FCT NOVA), Universidade Nova de Lisboa, 2829-516 Caparica, Portugal

**Keywords:** redox flow battery, membrane, surface modification, pore filling, active species crossover, capacity fade, energy efficiency, improved performance, long-term stability/durability

## Abstract

In this review, the state of the art of modified membranes developed and applied for the improved performance of redox flow batteries (RFBs) is presented and critically discussed. The review begins with an introduction to the energy-storing chemical principles and the potential of using RFBs in the energy transition in industrial and transport-related sectors. Commonly used membrane modification techniques are briefly presented and compared next. The recent progress in applying modified membranes in different RFB chemistries is then critically discussed. The relationship between a given membrane modification strategy, corresponding ex situ properties and their impact on battery performance are outlined. It has been demonstrated that further dedicated studies are necessary in order to develop an optimal modification technique, since a modification generally reduces the crossover of redox-active species but, at the same time, leads to an increase in membrane electrical resistance. The feasibility of using alternative advanced modification methods, similar to those employed in water purification applications, needs yet to be evaluated. Additionally, the long-term stability and durability of the modified membranes during cycling in RFBs still must be investigated. The remaining challenges and potential solutions, as well as promising future perspectives, are finally highlighted.

## 1. Introduction to Redox Flow Batteries

The intermittent nature of renewable energy sources has posed significant challenges to maintaining a steady power grid operation, resulting in temporal and spatial mismatches between electricity generation and consumption; thus, energy storage devices offer an effective solution to ensuring a reliable and efficient utilization of renewable energy [1,2].

Redox flow batteries (RFBs) are a type of electrochemical energy storage device that is currently attracting significant interest. These types of batteries are designed to store large amounts of energy for long periods, thus making them ideal for a range of applications, such as medium and grid-level energy storage applications and renewable energy integration [3,4].

**Figure 1:**
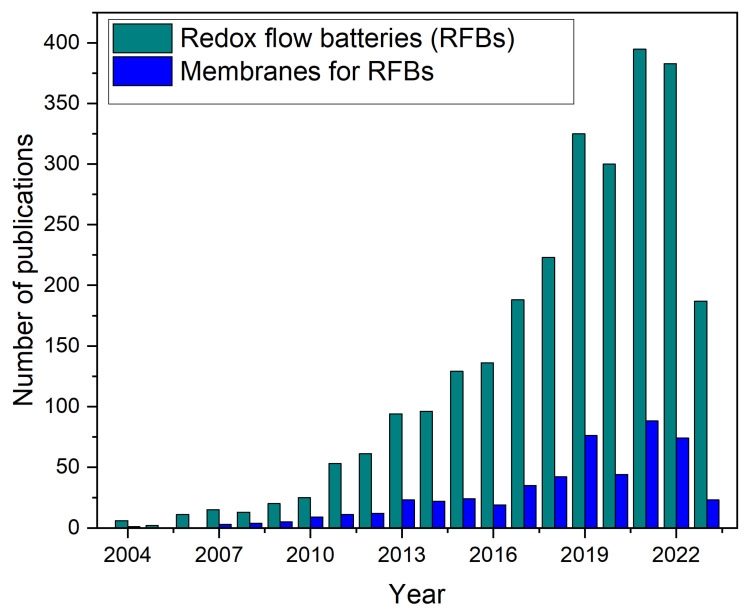
Number of publications on RFBs and membranes for RFBs per year from 2003 to 2023 mentioning the term “redox flow battery or RFB” for the RFBs and “redox flow battery or RFB” and membranes” for the membranes for RFBs as keywords. The data are derived from the Web of Science database (accessed on 10 July 2023).

Several research groups have focused on solving the challenges of RFBs in recent years, as evidenced by the growing number of published papers (Figure 1). The steady growth in scientific publications and citations over the last two decades indicates a significant interest in RFB technology as a promising option for energy storage. Quite a few excellent review papers have been published on RFBs research and development, mainly focusing on the redox active species [3,5,6,7]. Top of Form.

**Figure 2:**
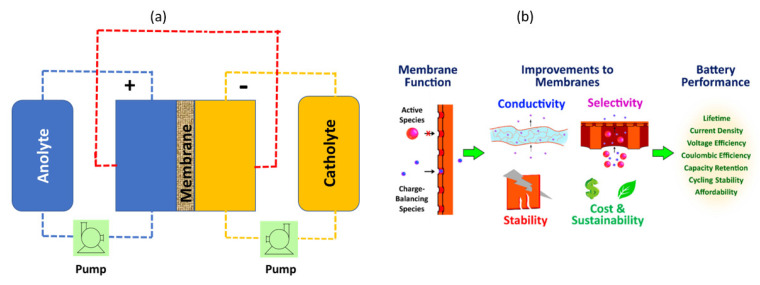
Schematic representation of a typical RFB (**a**) and the role of improved membranes in RFBs (**b**). (**b**) is taken with permission from [8]. Copyright 2021 American Chemical Society.

The key components of an RFB are the two types of redox-active species (one containing a positive electrolyte solution and the other a negative electrolyte solution), electrodes and a membrane that separates the two electrodes, as shown in Figure 2a. 

RFBs offer several advantages over other types of batteries, such as Li-ion and lead-acid batteries [6,7,9]. Their capacity can be easily increased by increasing the size of electrolyte tanks, making them ideal for large-scale energy storage systems. The power density can be adjusted independently to meet the needs of the customer or application by varying the number or size of stacks. RFBs also have a long-expected lifespan, as they can be charged and discharged several times (>10,000 cycles) without degradation. RFBs are safe to use, with no risk of fire, thus making them suitable for applications in which safety is a concern. Finally, they are mostly environmentally friendly when using non-toxic electrolyte solutions (such as water-soluble organic molecules) and are easily recyclable, with a lower environmental impact compared to that of other types of batteries. However, it must be noted that the overall environmental impact of RFBs, like other types of batteries, depends on various factors, such as the materials used in their production, the manufacturing process, their durability and their environmental impact upon disposal [10].

RFB performance can be evaluated in terms of energy density, power density, energy efficiency and cycling stability. These parameters are influenced by a variety of factors, such as the battery’s design, the nature of the active materials and the membrane used, among others. Table 1 summarizes the respective equations used to estimate these parameters. The theoretical energy density, which indicates the amount of charge stored, is primarily determined by three parameters.

i. Cell voltage (redox potential difference between the catholyte and anolyte),
ii. Solubility of the redox-active species and
iii. Number of electrons involved in the oxidation-reduction reactions, which defines the functioning of RFBs.

The power density of the battery depends on several parameters, including the voltage, cell internal resistance (the sum of the electrolyte, electrodes and connectors resistances), kinetics of the redox reactions and operating factors, including temperature, flow rate and flow field (uniformly distribute electrolytes into the electrode) used [11,12]. The cycling stability, on the other hand, depends on the (electro)chemical stability and degree of crossover of redox species.

**Table 1:** Main parameters in RFBs.

Parameters	Unit	Equation	Terms
Cell resistance (R)	Ω cm^2^	Area R = *R* (Ω) × *A* (cm^2^)	
Cell voltage (E_cell_)	Volt (V)	E_cell_ = E_positi–e_ − E_negative_	E_positive_ and E_negative_ are the potentials at the positive and negative electrodes, respectively.
Volumetric capacity (C)	Ah L^−1^	$C=\frac{m\;\times \;n\;\times \;F}{M\;\times \;V}$	*m* = mass, *n* = number of electrons, F = Faraday’s constant, M = molar mass and V = volume. E = energy density, U = E_cell_ − IR.
Theoretical energy density (E)	Wh L^−1^	*E* = *C* × *U*	
Coulombic efficiency (CE)	%	$\mathrm{CE}=\frac{t_{d}}{t_{c}}\times 100\%$	t_d_ is the discharging time, and t_c_ is the charging time. U_d_ = average discharging voltage, and U_c_ = average charging voltage when the same current was used for charging and discharging.
Voltage efficiency (VE)	%	$\mathrm{VE}=\frac{U_{d}}{U_{c}}\times 100\%$	
Energy efficiency (EE)	%	EE= CE×VE	
Current density	mA cm^−2^	$J=\frac{I}{A}$	I = discharge current, and A = active surface area of the membrane. U = output potential.
Power density	mW cm^−2^	Power density = J×U	
Capacity retention (CR)	%	$\mathrm{CR}=\frac{Q_{d,n}}{Q_{d,1}}$ × 100%	$\mathrm{Discharge}\;\mathrm{capacity}\;\mathrm{at}\;\mathrm{the}\;\mathrm{first}\;(Q_{d,1}$$)\;\mathrm{and}\;n\mathrm{th}\;\mathrm{cycle}\;(Q_{d,n})$.
$\mathrm{Permeability}\;\left(P\right)$ of active species	cm^2^ min^−1^	$P=\frac{V_{B}\times L}{A\times t}\ln\left(\frac{C_{A}}{C_{A}-C_{B}}\right)$	$L\;(\mathrm{cm})=\mathrm{membrane}\;\mathrm{thickness},\;A\;({\mathrm{cm}}^{2})=\mathrm{active}\;\mathrm{area},\;t(s)=\mathrm{elapsed}\;\mathrm{time},\;C_{A}\;\left(\frac{\mathrm{mol}}{{\mathrm{cm}}^{3}}\right)$ $=\mathrm{concentration}\;\mathrm{in}\;\mathrm{the}\;\mathrm{enrichment}\;\mathrm{side},\;C_{B}\;\left(\frac{\mathrm{mol}}{{\mathrm{cm}}^{3}}\right)$ $=\mathrm{concentration}\;\mathrm{in}\;\mathrm{the}\;\mathrm{deficiency}\;\mathrm{side}\;\mathrm{and}\;V_{B}=Volume\;of\;the\;deficiency\;side$
Diffusivity of active species (D)	cm^2^ min^−1^	$D=\frac{P}{K}$	K = partitioning coefficient. It denotes the amount of vanadium in the membrane in relation to the concentration of the bulk solution [13].

In RFBs, as shown in Figure 2b, the membrane plays an essential function as it separates the positive and negative electrolyte, preventing the cross-mixing of redox-active species, and conducts charge carrier ions (such as H^+^, HSO4^−^ in a vanadium (V) RFB) [8,14]. The membrane should ideally exhibit high ionic conductivity, a low crossover of active species, high mechanical strength, chemical stability and a low cost.

Porous membranes, modified porous membranes, anion-exchange membranes (AEMs) and cation-exchange membranes have been used in different RFB arrangements. However, there has been little focus on membrane development compared to other parts of the batteries, as can be seen from the number of published papers over the last 20 years in Figure 1.

We recently published a comprehensive review paper [15], that covered both theoretical models and experimental evidence on the transport of ions and active substances through the membranes, as well as the correlation between different membrane characteristics and cell performance. As discussed, porous membranes are often associated with high levels of active species crossover, while dense ion-exchange membranes currently utilized in RFBs have not been initially designed for this purpose and therefore exhibit low selectivity and efficiency [16].

To tackle this challenge, researchers have explored modified membranes as a potential solution, with a particular focus on the (surface) modification of porous membranes for RFBs. However, our previous review did not delve deeply into this area, and to the best of our knowledge, there is currently no review article published in the open literature on the latest advancements in the fields of modified membranes for RFBs. Such membranes have been found to be promising in terms of selectivity and cost for VRFBs [17] and zinc (Zn)-based RFBs [18,19]. Herein, the state-of-the-art surface-modified membranes in RFBs are presented and critically discussed. The relationship between different membrane modification strategies and their impact on cell performance is discussed. The remaining challenges and potential solutions to improve the membrane performance are also outlined.

The review is organized as follows: Section 1 gives a brief introduction on RFBs. Section 2 begins with a discussion of the common membrane modification techniques. Section 3 then discusses the recent advances in surface modified membranes used in RFBs. The remaining challenges, as well as potential strategies for overcoming them are discussed in Section 4. Finally, a conclusion and perspective for employing appropriately modified membranes for high-performance RFB systems are provided.

## 2. Membrane Modification Techniques

The performance of membranes is significantly influenced by the properties of their surface; as in most electrochemical systems, the membrane surface is in direct contact with the electrolyte. Therefore, altering the membrane surface properties can have a significant effect on their performance through changes of the surface chemistry, surface charge, roughness and hydrophilicity/hydrophobicity. For instance, the surface modification of commercial membranes enables the tuning of desirable properties, like flux and selectivity for specific separation processes [20].

**Figure 3:**
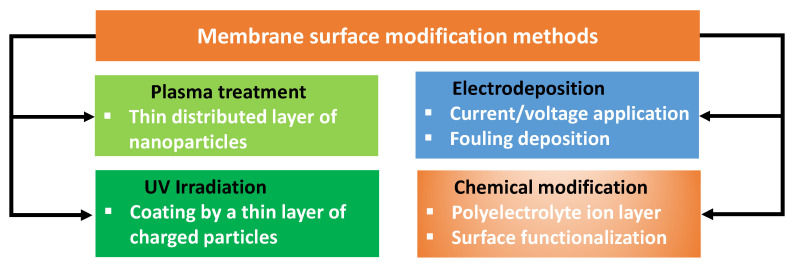
Overview of the methods for membrane modification.

Surface modifications of membranes are widely employed in the field of water treatment, and it is in this domain that one can find the majority of information pertaining to modification techniques. Surface modification of membranes can involve adding a coating or functionalizing the surface to endow desired properties to a membrane. This can be performed by methods like chemical treatment, plasma/UV treatment or physical deposition (Figure 3, Table 2). Some of the most common techniques used for surface modification membranes are briefly discussed below.

### 2.1. Plasma Treatment

**Figure 4:**
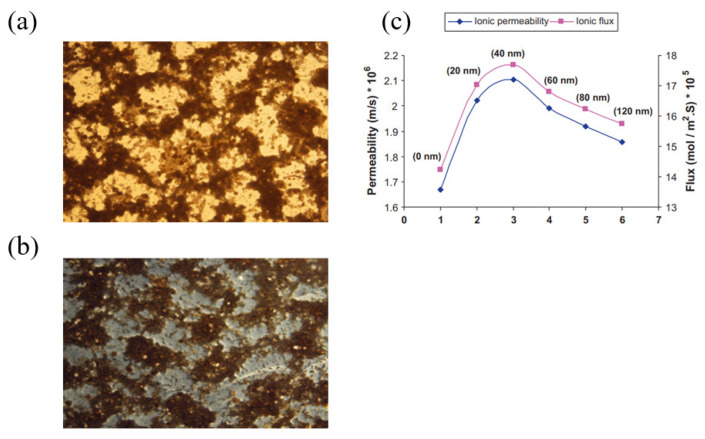
SEM images of membranes: unmodified membrane (**a**), modified membrane with 40 nm Ag nanolayer (**b**), ionic permeability and flux of unmodified membrane and modified membranes with various deposited nano-silver layer thickness (nm) on the membrane surface (**c**). Figures are taken with permission from [24]. Copyright 2010 Elsevier.

Plasma treatment has become an important surface modification method as a result of its relatively straightforward procedure, which is fast, solvent-free and environmentally friendly. In surface plasma modification, gases like oxygen, nitrogen and hydrogen are used to react and modify the substrate’s surface, changing its characteristics, including wettability, printability and adhesion [21]. The technique can be generally used for surface cleaning, activation, crosslinking, etching or often a combination of these effects [22]. Plasma treatment improves surface energy and enhances the adhesion properties for technical applications [23], and it is commonly used for membrane surface modification. As an example, S.M. Hosseini et al. [24] reported the deposition of ultrathin silver layers on the surface of polyvinylchloride (PVC)/Styrene-Butadiene-Rubber (SBR) blend heterogeneous cation-exchange membranes using argon plasma treatment. The modification with silver nanoparticles improved several properties, including the surface charge density, transport number, permselectivity, electrical conductivity, ionic permeability, ionic flux and current efficiency, as shown in Figure 4. The best-performing membranes were observed to be the ones with an Ag nanolayer thickness of 40 nm. In another study, Zendehnam et al. [25] found that initial deposition of a 5 nm Ag nanolayer on the membrane surface led to a decrease in the membrane potential, surface charge density, transport number and selectivity. However, increasing the nanolayer thickness from 5 to 30 nm resulted in an increasing trend for these properties. The deposition of Ag nanoparticles/nanolayer through plasma treatment presented a challenge as the membrane charge density and selectivity initially declined but showed improvement with greater nanolayer thickness.

The rigorous temperature requirements necessary for plasma formation is a challenge of plasma modification. This method has been the least studied in the past years due to its increased complexity and the greater robustness of the produced membranes [26]. It also requires high-cost equipment and consumes significant energy related to maintaining the desired temperature and pressure conditions. In spite of the high overall cost and complex production, the intrinsic properties of plasma treatments such as fast reaction time, waste-free processes, high versatility and facilitation of the bond formation between the membrane and the modifying agents makes it an effective strategy to enhance other modification methods.

### 2.2. UV Irradiation

UV irradiation is a cost-effective, efficient and non-contact method for modifying surfaces. It is a physical method of modifying a membrane’s surface by exposing it to UV light. It offers several advantages, including effectiveness, economic viability and a streamlined process with minimal steps. The UV radiation generates reactive species and free radicals on the membrane surface, which can react with the surface and change the membrane properties. This technique is commonly employed to improve the membrane’s hydrophilicity, permeability and anti-fouling characteristics [27]. In a study by Abdi et al. [28], PES-based ultrafiltration membranes were modified by inherently hydrophilic hydrous ferric oxide particles using UV irradiation. The resulting membranes exhibited super-hydrophilic properties with excellent performance in the separation of oil-water emulsions, as evidenced by their high-water flux and low fouling. Additionally, the membranes demonstrated a high flux recovery ratio, further highlighting their efficiency in oil–water emulsion separation. Güler et al. [29] reported commercial AEM modified by coating with a thin negatively charged layer formed through the copolymerization of 2-acryloylamido-2-methylpropanesulfonic acid (AMPS) as the active polymer and N,N-methylenebis(acrylamide) as the crosslinker, utilizing UV irradiation. The resulting membrane displayed enhanced monovalent-ion selectivity toward Cl^−^ ions against SO_4_^2−^ ions, increased hydrophilicity, and showed effective resistance against fouling caused by organic substances. Challenges in UV irradiation modification include high equipment requirements and the risk of surface damage if proper control measures are not implemented [30].

### 2.3. Electrodeposition

Electrodeposition is a method of depositing a layer on a membrane surface using an electrical field. The process involves placing the membrane in a cell with a solution and electrodes and then applying electrical potential to the electrodes. This causes the modifier to be attracted to the electrode with an opposite charge [31]. It is a process that occurs with a high deposition rate, and it is easy to regulate, cost-effective and portable [32]. Zhao et al. [33] employed a layered surficial electro-deposition method as a membrane modification technique using polyethyleneimine, with the aim to enhance the selectivity for monovalent cations and prolong membrane lifetime. The outcome demonstrated that the modification method successfully restored the ion-exchange groups of the commercial Selemion^®^ CSO membranes modified with polyethyleneimine, resulting in high permselectivity. They also used electrodeposition to enhance the permselectivity of an anion-exchange membrane by alternatively depositing poly(sodium 4-styrene sulfonate) (PSS) and hydroxypropyl trimethyl ammonium chloride chitosan (HACC) onto the membrane surface. In comparison to the original commercial AEM, (PSS/HACC) N bilayers greatly enhanced anion selectivity. The results of electrodialysis experiments demonstrated an increase in monovalent selectivity of ${\mathrm{Cl}}^{-}/{\mathrm{so}}_{4}^{2-}$ from 0.66 to 2.90 and an improvement in separation efficiency from −0.19 up to 0.28 when nine PSS/HACC bilayers were used [34]. It may be challenging to control deposited layers’ thickness and uniformity using this electrodeposition method. Pan et al. [35] prepared a monovalent selective anion-exchange membrane by covalently electrodepositing polyethyleneimine (PEI) on the surface of a partially quaternized poly (phenylene oxide) (QPPO) AEM. The modification of the heterogeneous AEMs resulted in an increase in the monovalent permselectivity of the membrane for chloride ions (Cl^−^) over sulfate ions (SO_4_^2−^) from 0.79 to 4.27. This increased permselectivity was attributed to a reduction in the sulfate-ion leakage rate from 39.6% to 19.4%.

Li et al. [36] investigated the modification of AEMs using graphene oxide (GO) through electrodeposition. Their findings demonstrated that higher GO concentrations (0.1–0.5 g/L) and lower NaCl concentrations (0.01 M) increased hydrophilicity and negative charge density. However, higher NaCl concentrations led to reduced modification effects due to competitive migration of Cl^−^ ions. The GO-modified AEMs showed smoother surfaces, higher hydrophilicity and negative zeta potential compared to pristine AEMs. The study suggests that GO-modification can improve AEM properties without affecting desalination performance. The challenge with the modification method used in this study is the potential hindrance of electrodeposition of graphene oxide (GO) on the AEMs due to the competitive migration between GO and chloride (Cl^−^) ions in higher concentrations of NaCl, the supporting electrolyte. Therefore, careful control of the NaCl concentration is necessary to optimize the electrodeposition process of GO on the AEMs.

The electrodeposition method generally creates difficulty in controlling the thickness and uniformity of the deposited polyelectrolyte layer. The optimization of several parameters, including temperature, deposition time, current density or applied voltage, pH level, as well as electrode materials, is essential to achieve the desired thickness of layer and uniform coating by electrodeposition [37]. Another concern is the possibility of damaging the membrane surface during the electrodeposition process and the stability and durability of the electrodes used in electrodeposition, as they play a vital role in this modification technique. Moreover, electrodeposition encounters limitations in terms of the range of materials that can be deposited. The deposited layer may also exhibit instability under certain conditions, which can negatively impact membrane performance [31].

### 2.4. Chemical Modification

**Figure 5:**
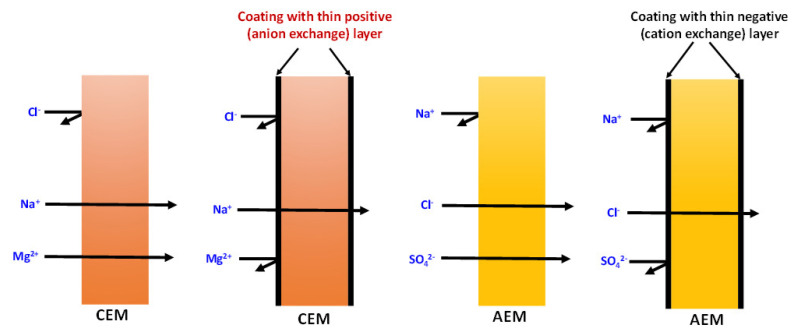
Modification of ion-exchange membranes toward monovalent selectivity by coating the surface with a thin, charged layer using chemical methods.

Chemical modification of a membrane surface is an appealing method for introducing favorable surface characteristics while maintaining the desired properties of the membrane such as mechanical strength, chemical resilience and a target membrane structure. A typical strategic scheme of chemical modification, for instance, to enhance monovalent selectivity by introducing a thin, oppositely charged layer over the surface of an ion-exchange membrane, is presented in Figure 5. This process involves the formation of covalent or ionic bonds between the modifier and the membrane surface, resulting in a more durable modification. The modified surface properties are less likely to change over time due to the creation of chemical bonds, making it suitable for long-term operations [38].

Zhang et al. [39] employed chemical modification to enhance the ion selectivity of a polyacrylonitrile (PAN) membrane by assembling silica on its surface for VRFB applications. The resulting modified membranes exhibited enhanced ion selectivity maintaining good ion conductivity, thus making them a promising substitute for Nafion in VRFB applications. This method offers a universal and efficient approach for fabricating high-performance porous membranes suitable for VRFB separators.

Hwang et al. [19] coated a Celgard^®^ 5550 membrane with a polyelectrolyte ion layer to reduce the crossover of zincate ions in a rechargeable Zn-air battery. The ion-selective layer was made of an anion-exchange polymer prepared via the free radical polymerization of selected monomers. The coating improved the selectivity of the membrane, resulting in a longer lifespan for the battery compared to one using a non-modified membrane. The challenge encountered in this study’s modification method is the need for precise control over the thickness and uniformity of the copolymer coating on the Celgard membrane. This difficulty can affect battery performance by causing inconsistent anionic transfer across the separator and leading to elevated Zn crossover. Additionally, the synthesis of the copolymer requires the careful management of reaction conditions to achieve the desired functionality and structural integrity of the resulting material.

The use of conducting polymers for membrane modification allows for reducing the loss in membrane conductivity during membrane modification. Tufa et al. [40] employed chemical modification using polypyrrole (PPy)/chitosan (CS) composite for the surface modification of cation-exchange membranes (CEMs). The monovalent selectivity of the membranes exhibited a three-fold improvement compared to unmodified membranes, with an increase in the open-circuit voltage (OCV) up to 20%. The modified membranes also showed power densities in the range from 0.6 to 1.5 W/m MP (MP: membrane pair), which represented a significant improvement of over 42% compared to the original membranes. This research sets aside perspectives in utilizing conducting polymers such as polyaniline and poly (p-phenylene sulfide) to design highly selective and conductive membranes with optimization of the surface modification.

The demand for more environmentally friendly membranes has significantly grown in recent years [41]. Bio-based polymers offer an opportunity to enhance sustainability in membrane technology by serving as alternative materials for the production of environmentally friendly polymeric membranes [42]. Cellulose and chitosan are popular choices for producing environmentally friendly polymeric membranes that find application in a wide range of membrane separation processes [43]. Keraani et al. [44] developed an eco-friendly surface functionalization method for PES membranes using bio-sourced aryl diazonium salts. The innovative approach involves grafting aryl radicals, UV irradiation and bio-based monomers to create partially bio-sourced functionalized membranes. The functionalized membranes exhibited improved rejection of charged molecules and enhanced antifouling properties. This concept aligns with European union’s “Green” priorities and can be extended to the surface modification of other membranes as well. In Zn slurry-air flow batteries, the key challenge is the undesirable crossover of active species. Membrane modification has been reported as one strategy to overcome this problem. Tsehaye et al. [18] coated ion-selective ionomer containing modified poly (phenylene oxide) (PPO) and N-spirocyclic quaternary ammonium monomer on Celgard^®^ 3501 and crosslinked via UV irradiation (PPO-3.45+3501). The results showed that the PPO-3.45+3501-based cell produced a peak power density of 66 mW cm^−2^ and 18-times-lower zincate ion crossover compared to pristine membrane.

Chemical modification of membranes has received significant attention in membrane research and is currently a vibrant and highly researched area. Despite the extensive knowledge gained and various strategies employed in membrane chemical modification, obtaining a deep understanding of the fundamental principles of modification, the relationship between surface modifying agents and membrane surfaces, as well as achieving durable antifouling properties, remains challenging. There are still concerns regarding the enduring stability, uniformity, shelf-life, cost-effectiveness, scalability and leachability of the modifying agent from the membrane surface in the long term [45].

Overall, membrane surface modification methods have a huge potential in improving some of the desirable membrane properties like selectivity and stability. For instance, in RFBs, the use of appropriately modified membranes can make it possible to mitigate crossover issues and significantly enhance ion transport efficiency, thereby addressing challenges like low energy efficiency and membrane instability. However, how to develop more effective modification techniques and determine the best-performing modification materials is the prospective research question.

**Table 2:** Summary of selected membrane surface modification methods.

Modification Materials	Membrane Modification Methods	Controlled/Improved Properties	Outcome/Performance	Ref.
Silver nanoparticles,	Plasma treatment	Surface charge density Permselectivity Membrane electrical conductivity Flux and current efficiency	Membrane with 40 nm thickness demonstrated suitable performance compared to unmodified membrane	[24]
Fe-Ni oxide nanoparticles and Ag nanolayer.	Plasma treatment	Physicochemical characteristics Antibacterial characteristics	Increased membrane smoothness, increased ionic flux, good ability of membranes for *E. coli* removal	[25]
Hydrous ferric oxide particles	UV irradiation	Flux Fouling property	Increased flux, low fouling	[28]
AMPS MBA	UV irradiation	Monovalent-ion selectivity antifouling potential gross power density	Increased monovalent-ion selectivity, sufficient antifouling potential	[29]
Polyethyleneimine	Electrodeposition	Monovalent cations selectivity Llifetime	Increased permselectivity, increased lifetime of the membranes	[33]
PSS HACC	Electrodeposition	Monovalent selectivity Separation efficiency	Increased monovalent selectivity from 0.66 to 2.90, increased separation efficiency from −0.19 to 0.28	[34]
PEI solution	Electrodeposition	Permselectivity Hydrophilicity of the membrane surface	Increased permselectivity, increased hydrophilicity of the membrane surface	[35]
Graphene oxide	Electrodeposition	Membrane roughness Hydrophilicity Fouling properties	Smoother surface, increased hydrophilicity, iIncreased fouling resistance	[36]
PSS PAAS Poly (vinyl sulfonic acid), Sodium salt) (PVS)	Electrodeposition	Physicochemical properties antifouling performance desalination performance	Increased antifouling property and best with PVS	[46]
Silica nanoparticles	Chemical modification	Ion selectivity	Increased ion selectivity	[39]
SPVA Glutaraldehyde	Chemical modification	Water flux Salt rejection Fouling resistance	Increased salt rejection rate (99.18%), Increased flux recovery above 95%, Increased antifouling resistance	[47]
EBIH and BMA monomers	Chemical modification (free radical polymerization)	Zincate crossover Durability Battery life	Reduced Zincate crossover, increased durability and increased battery life	[19]
Polypyrrole (PPy)/chitosan (CS)	Chemical modification	Ion selectivity Power density	Increased power density from 0.23 W/m^2^ to 0.45 W m^−2^, increased ion selectivity	[40]
PPO N-spirocyclic quaternary ammonium monomer	Chemical modification	Zincate ion crossover Power density	Reduced zincate ions crossover, increased peak power density to 66 mW cm^−2^	[18]

The properties and performance of modified membranes in different RFB chemistries are discussed in the following section.

## 3. Recent Advances in Modified Membranes for RFBs

Our extensive literature search suggests that the topic of membrane surface modification has still not been thoroughly explored in the RFB domain. There are only a few research papers available on this subject, with most of the works focusing on membranes for VRFB, which has been relatively more studied and is therefore discussed first. Modified membranes used in Zn-based RFBs and aqueous organic RFBs (AORFBs) are discussed next. A comprehensive summary in the form of a table is also included at the end of each battery chemistry.

### 3.1. Modified Membranes for VRFBs

The electrolyte tanks in VRFBs contain 2–4 M H_2_SO_4_ solutions of electrochemically reversible redox couples: VO^2+^/VO_2_^+^ as a positive active material and V^2+^/V^3+^ as a negative active material. One major problem encountered by the state-of-the-art ion-exchange membranes used in VRFB (mainly Nafion) is their relatively high vanadium ion crossover, which results in a capacity fade during long-term operation and leads to self-discharge when the battery is in storage [48,49]. Various methods, such as radiotracer permeation tests [50], are used to determine vanadium ion (VO^2+^) crossover through membranes.

Several membrane modification techniques, such as composite membranes (incorporation of inorganic particles into a polymeric membrane) [51,52], polymer blending [53] and interfacial polymerization [54], are used to lower vanadium ion crossover. These techniques are discussed in detail below.

A thin cationic charged layer was formed, using polyethylenimine polyelectrolyte and chlorosulfonyl, on a Nafion 117 membrane surface using interfacial polymerization [54]. The Nafion-PEI-2.5 membrane, prepared by soaking a Nafion membrane in 2.5% PEI aqueous solutions, exhibited much lower VO^2+^ ion crossover (5.23 × 10^−7^ cm min^−1^ vs. 36.55 × 10^−7^ cm min^−1^), while its resistance increased from 1.06 to 1.24 Ω cm^2^ following the modification compared to the pristine Nafion membrane. It seems that the PEI-based cationic charged layer acts as an effective barrier to the vanadium ion crossover via a Donnan exclusion effect [55]. As a result, the modified membrane enabled a higher Coulombic efficiency (CE) over the Nafion membrane (96.2% vs. 93.8%) at a 50 mA cm^−2^ current density in a VRFB single cell. On the other hand, Nafion-PEI-5 showed 97.3% CE under the same testing conditions; however, its resistance was reported to be 1.34 Ω cm^2^, indicating the need for optimizing the coat thickness to achieve the best trade-off between an acceptable resistance and avoided (minimized) vanadium ions crossover. A detailed comparison of the properties of the membranes and their battery test performance is provided in Table 3.

In another study, aiming at preventing the crossover of vanadium crossover in VRFB, three different membrane surface modification methods (electrolyte soaking, oxidation polymerization by FeCl_3_ and polymerization by electrodeposition) were employed to modify Nafion 117 using pyrrole [49]. The electrodeposition method (performed at 0.025 mA cm^−2^ and 0 °C for 1 h) was identified to be the most appropriate among them, reducing the vanadium ion crossover from 2.87 × 10^−6^ cm^2^ min^−1^ (Nafion 117) to 5.0 × 10^−7^ cm^2^ min^−1^ (modified Nafion 117).

Pore filling (in case of porous membranes) with a polyelectrolyte was reported to be an effective method to minimize the crossover of vanadium ions in VRFB by the research group of M. Skyllas-Kazacos [56]. In this study, a microporous separator (Daramic, ~100 nm pore size) was first impregnated with an ion-exchange resin (Amberlite CG400) by immersing the membrane in the polyelectrolyte solution, followed by crosslinking using divinyl benzene. The VRFB employing the modified membrane delivered 94% CE and 81% EE at 40 mA cm^−2^ for 1650 charge–discharge cycles. The improvement in CE was attributed to the blocking of the pores of the membrane by the ion-exchange resin (<20 nm pore size) and the cross-linking.

**Figure 6:**
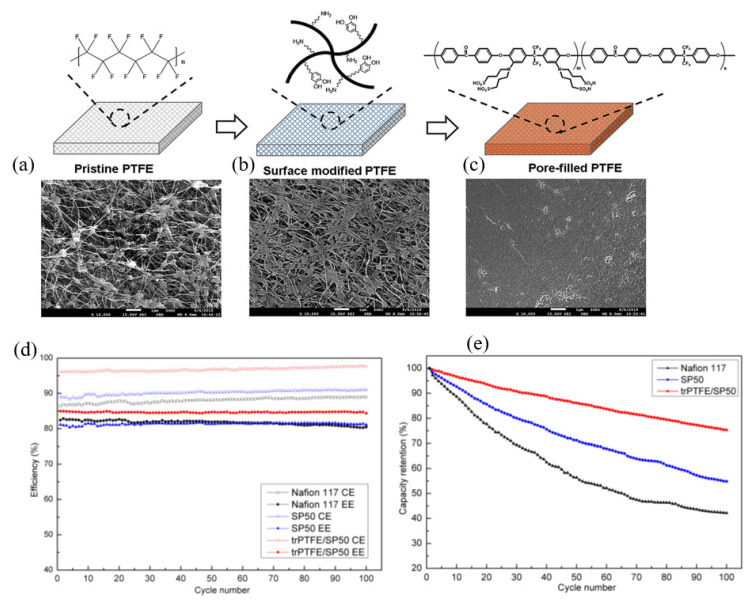
Schematic representation of the modification process with their respective surface SEM images of (**a**) the pristine PTFE, (**b**) trPTFE, (**c**) trPTFE/SP, energy efficiency (**d**) and capacity retention (**e**) VRFB single cell employing the different membranes at 40 mA cm^−2^ for 100 cycles. Taken with permission from [57]. Copyright 2020 Elsevier.

A porous polytetrafluoroethylene (PTFE) substrate (from Donaldson Korea) was impregnated with sulfonated poly(arylene ether ketone) (SP) by Ahn and Kim [57]. The SP solution was poured on the porous PTFE, and a doctor blade was used to cast and fill the pores with SP solution while maintaining the uniform thickness, as schematically shown in Figure 6. Modified membranes with varying thicknesses (25 μm for trPTFE/SP30, 27 μm for trPTFE/SP40 and 24 μm for trPTFE/SP50 membranes) were fabricated. The pristine membrane before the pores-filling process was only 12 μm thick. The prepared SP-filled PTFE membrane (trPTFE/SP) was tested in VRFB and compared with the pristine and Nafion membranes. The pore-filling process resulted in a slight reduction of the membrane proton conductivity (by 10%), but it significantly decreased the permeability of vanadium ions by about five times. The SP membranes had a VO^2+^ ion permeability ranging from 1.37 × 10^−7^ cm^2^ min^−1^ to 4.21 × 10^−7^ cm^2^ min^−1^, which was 15-times lower than that of Nafion117, whose vanadium permeability was 20.28 × 10^−7^ cm^2^ min^−1^. This resulted in a high CE (>96%) and energy efficiency (EE) (~84%) during 100 cycles. Figure 6 shows the CE, EE and capacity retention of a VRFB single cell employing the modified and reference membranes at 40 mA cm^−2^ for 100 cycles.

Recently, the same research group [58] prepared an AEM by filling the pores of PTFE support with poly(arylene ether ketone) with imidazole (imidazolium grafted poly(arylene ether ketone), abbreviated as PAPI). Catechol and polyethyleneimine were first co-deposited on the hydrophobic PTFE, making it more hydrophilic, followed by impregnation of the imidazole molecules. The modified membrane had a vanadium ion permeability 3times and 10-times lower than those of FAP450 and Nafion 117 membranes, respectively. The VRFB employing the prepared membrane (PTFE/PAPI 2.5) delivered a high CE (96.5%) and excellent EE (85%) with 200 cycles at 40 mA cm^−2^, making the pore-filling of a porous substrate with an ionomer a promising strategy for preparing membranes for VRFB applications.

Another class of modified membranes used in VRFBs are organic–inorganic composite membranes. The inorganic materials are either mixed with the polymer or coated as a thin layer on top of the (ion-exchange) membrane. The recent progress on this topic is discussed in the following text.

**Figure 7:**
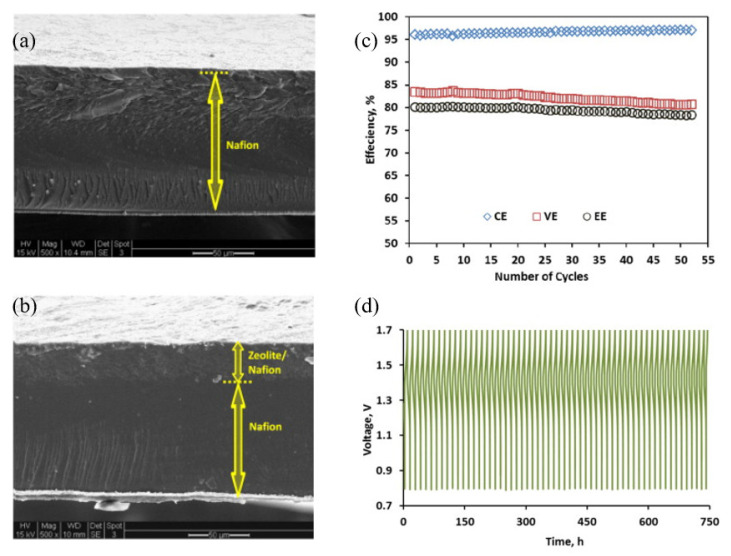
Cross-section images of recast pure Nafion film (**a**), ZNM-5 (**b**) and ZNM-5-based VRFB performance, battery efficiencies as a function of cycle number (**c**) and charge–discharge curves as a function of operating time (**d**) at 40 mA cm^−2^. Taken with permission from [59]. Copyright Elsevier 2015.

Yang et al. [59] prepared a composite membrane composed of Nafion base and a thin layer (<30 μm) of silicalite nanoparticles. It was reported that the nonionic silicalite nanoparticle content increased the composite membrane’s proton selectivity and electrical resistance. The cross-section images of the composite membrane containing a total zeolite content of 5 wt.% (ZNM-5, the number 5 refers to the thickness increase recorded compared to a recast pure Nafion) and pure recast Nafion-117 are shown in Figure 7a,b. The VRFB cell with the ZNM-5 membrane outperformed the Nafion-117-based cell in terms of CE, VE and EE. This was attributed to the improved proton selectivity and reduced resistance of the former membrane. The ZNM-5-based cell was cycled for more than a month at 40 mA cm^−2^. The reported efficiency values (CE, VE and EE), as a function of the cycle number, and charge–discharge curves of the battery are presented in Figure 7c,d. The VRFB with the modified membrane demonstrated a good stability of EE over the 30-day test period. Additionally, the membrane’s morphology remained unchanged after the battery test.

Another effective method for preparing membranes with reduced crossover of vanadium ion is the sol–gel modification of commercial ion-exchange membranes. The preparation and use of sol–gel-modified Nafion membranes in VRFBs have been reported in the literature [60,61,62,63]. A silica nanocomposite AEM was also prepared via an in situ sol–gel reaction [64]. The silica nanoparticle-incorporating membrane was reported to be more effective in preventing the permeation of vanadium ions. The prepared membranes exhibited a vanadium ion permeability of about 20% lower than that of the pristine AEM and one order of magnitude lower than that of the commercial Nafion CEM, resulting in a high CE (92%) of the modified membrane-based VRFB. Detailed membrane properties and battery performance data are provided in Table 3.

**Figure 8:**
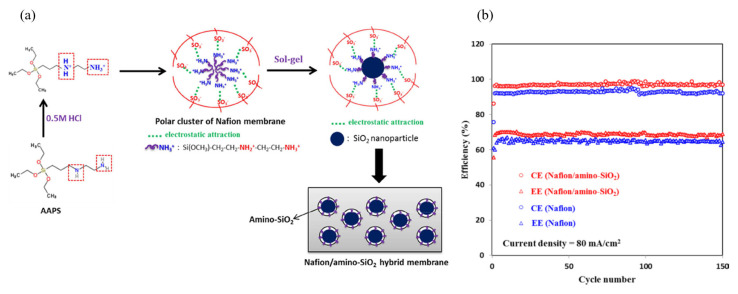
Schematic representation of the preparation of Nafion/amino-SiO_2_ hybrid membrane (**a**), and VRFB performance of the Nafion and modified membranes as function of cycle numbers (**b**). Taken with permission from [65]. Copyright 2015 Elsevier.

In almost all cases, modification of porous membranes with inert polymer or inorganic materials has resulted in a reduced vanadium ion crossover. On the other hand, the ionic conductivity of the membranes has also been reduced. Aiming at optimizing this trade-off issue between membrane ion selectivity and conductivity, Lin et al. [65] prepared a Nafion/amino-SiO_2_ hybrid membrane by incorporating SiO_2_ nanoparticles into Nafion membranes. The amino-SiO_2_ was incorporated into the membrane via in situ sol–gel reactions of N-(2-aminoethyl)-3-aminopropyltrimethoxysilane. The process is schematically depicted in Figure 8a. A comparison of the properties between Nafion and Nafion/amino-SiO_2_ hybrid membranes and their VRFB performance is shown in Figure 8b and Table 3. The prepared composite membrane exhibited reduced VO_2_^+^ and VO^2+^ ion permeability, about 27% and 31% of the pristine Nafion value, respectively. The reduction in the permeability of the two species involved in VRFBs through the modified membrane was attributed to the amino-SiO_2_ nanoparticles filled into the polar clusters of the Nafion membrane. Interestingly, the modified membrane exhibited better ion selectivity while keeping the ionic conductivity almost unchanged. A recently published paper [66] discussed different approaches to develop membranes with enhanced ion selectivity specifically designed for all-VRFBs.

Similarly, graphene oxide (GO) was used to prepare a composite membrane with reduced vanadium ion permeability, as well as improved mechanical stability and chemical resistance. The addition of GO to the recast Nafion caused the water channels in the composite membrane to shrink because the sulfonated acid groups in the Nafion matrix interacted with the oxygen-containing groups in GO [67]. The randomly distributed GO twisted the water channels, which made it difficult for the VO^2+^ ion to move through them. As a result, the vanadium permeability of the membrane was cut in half, and there was a slight reduction in the proton conductivity. The GO/Nafion-based VRFB showed higher CE (96% vs. 91%) and EE (85% vs. 80%) than the recast Nafion-based cell at 80 mA cm^−2^ [67]. The performance of GO-modified membranes in VRFBs has been reviewed in more detail elsewhere [68].

**Table 3:** Vanadium permeability and VRFB performance of the modified membranes.

Modification	Membrane	Property	VO^2+^ Ion Permeability	Battery Performance	Ref.
Interfacial polymerization	Nafion-PEI-2.5	196 µm-thick 1.24 Ω cm^2^ 0.89 mmol g^−1^ (IEC *)	5.23 × 10^−7^ cm min^−1^	CE: 96.2% VE: 88.4% EE: 85.1% (at 50 mA cm^−2^)	[54]
	Nafion-PEI-5	208 µm-thick 1.34 Ω cm^2^ 0.87 mmol g^−1^ (IEC)	1.70 × 10^−7^ cm min^−1^	CE: 97.3% VE: 83.3% EE: 81.1%	
	Nafion 117	175 µm-thick 1.06 Ω cm^2^ 0.91 mmol g^−1^ (IEC)	36.55 × 10^−7^ cm min^−1^	CE: 93.8% VE: 90.7% EE: 85.0%	
Thin inorganic layer	ZNM-5	~120 μm 0.55 Ω (*R_m_*)	***α_H_***_+/***V*****4**+:_ ~23	CE: >95% EE: 77% (at 60 mA cm^−2^)	[59]
	ZNM-15	~130 μm 2.23 Ω (*R_m_*)	***α_H_***_+/_***_V_***_**4**+:_ ~46	CE: >95% EE: ~57%	
	Nafion-117	~183 μm 0.81 Ω (*R_m_*)	***α_H_***_+/_***_V_***_**4**+:_ ~19	CE: >95% EE: 65%	
Deposition of polypyrrole	PHB12 sample (Via electrodeposition of Nafion 117 at 0.025 mA cm^−2^ and 0 °C for 60 min)	7.83 mS cm^−1^ H^+^ conductivity	0.54 × 10^−6^ cm^2^ min^−1^	NA	[49]
	A9 sample (via 9 h electrolyte soaking)	0.733 mmol g^−1^ IEC 3.30 mS cm^−1^ H^+^ conductivity	1.02 × 10^−6^ cm^2^ min^−1^		
	P2 sample (Via polymerisation by FeCl_3_)	3.47 mS cm^−1^ H^+^ conductivity	1.48 × 10^−6^ cm^2^ min^−1^		
	Nafion 117	0.861 mmol g^−1^ IEC 8.58 mS cm^−1^ H^+^ conductivity	2.87 × 10^−6^ cm^2^ min^−1^		
Sulfonation (of AEMs)	Sulfonated Selemion AMV	2.45 Ω cm^2^	V(IV) diffusivity: 38.5 × 10^5^ cm min^−1^	CE: 96.0% VE: 82.5% EE: 79.2% (100 cycles at 30 mA/cm^2^)	[69]
	Selemion AMV	2.80 Ω cm^2^	V(IV) diffusivity: 0.32 × 10^5^ cm min^−1^	CE: 98.5% VE: 81.4% EE: 80.2% (100 cycles at 30 mA/cm^2^)	
	Modified New Selemion (PSSS ** Selemion, 2 h)	1.25 Ω cm^2^	V(IV) diffusivity: 4.11 × 10^5^ cm min^−1^	CE: 100% VE: 83.4% EE: 83.4% (50 cycles at 40 mA/cm^2^)	
	New Selemion (Type 2)	0.98 Ω cm^2^	V(IV) diffusivity: 11.6 × 10^5^ cm min^−1^	CE: 98.6% VE: 87.5% EE: 86.3% (50 cycles at 50 mA/cm^2^)	
Pore filling with ion-exchange resin	Amberlite CG400-filled Daramic	20 nm pore size Less than 3 Ω cm^2^		CE: >90% 1650 cycles	[56]
	Daramic microporous	100 nm pore size		CE: 77%	
Pore filling (impregnating) of sulfonated poly(arylene ether ketone) (SP)	trPTFE/SP50	24 μm-thick 1.8 meq g^−1^ IEC 46 mS cm^−1^ H^+^ conductivity	4.21 × 10^−7^ cm^2^ min^−1^	CE: >96% EE: 84% (at 40 mA cm^−2^ for 100 cycles)	[57]
	Porous polytetrafluoroethylene (PTFE) substrate membrane	NA	20.28 × 10^−7^ cm^2^ min^−1^	NA	
	Nafion 117	0.9 meq g^−1^ IEC 50 mS cm^−1^ H^+^ conductivity	20 × 10^−7^ cm^2^ min^−1^	CE: 90% EE: ~82% (at 40 mA cm^−2^ for 100 cycles)	
Ionomer-filling of PTFE	PTFE/PAPI 2.5	1.51 meq g^−1^ IEC 42 μm	2.08 × 10^−7^ cm^2^ min^−1^	200 cycles, CE: 96.5% EE: 85%,	[58]
Silica nanocomposite AEM	Silica modified AEM (AEM Sol–gel 30 s)	60 μm 5.60 wt.% silica 1.13 mmol g^−1^ IEC 1.088 Ω cm^2^	4.24 × 10^−7^ cm^2^ min^−1^	CE: ~92%, EE: ~73% (40 mA cm^−2^)	[64]
	Pristine AEM (Fumasep FAP)	60 μm 1.16 mmol g^−1^ IEC 0.7 Ω cm^2^	5.24 × 10^−7^ cm^2^ min^−1^	CE: ~89%, EE: ~75% (40 mA cm^−2^)	
	Nafion 115 CEM	127 μm 0.91 mmol g^−1^ IEC 0.987 Ω cm^2^	1.62 × 10^−6^ cm^2^ min^−1^	CE: ~87%, EE: ~71% (40 mA cm^−2^)	
A hybrid membrane of Nafion/amino-silica (amino-SiO_2_)	Nafion/amino-SiO_2_ hybrid membrane	188 μm 1.05 mmol g^−1^ 3.45 Ω cm^2^	2.32 × 10^−7^ cm^2^ min^−1^	CE: >96% EE: ~70%, (80 mA cm^−2^ for 100 cycles)	[65]
	Pristine Nafion 117	186 μm 0.96 mmol g^−1^ 3.36 Ω cm^2^	8.65 × 10^−7^ cm^2^ min^−1^	CE:~92% EE: ~68%, (80 mA cm^−2^ for 100 cycles)	
Graphene-oxide modified membrane	Nafion/GO	70 μm, 0.88 mmol g^−1^ 29 mS cm^−1^ H^+^ conductivity	~12 × 10^−7^ cm^2^ min^−1^	CE: 96%, EE: 85% (80 mA cm^−2^)	[67]
	Recast Nafion	58 μm, 0.85 mmol g^−1^ 31.5 mS cm^−1^ H^+^ conductivity	~22 × 10^−7^ cm^2^ min^−1^	CE: 91%, EE: 80% (80 mA cm^−2^)	
Cation-exchange ionomer/(WO_3_) hybrid membrane	Nafion/(WO_3_)_0.587_	wt% of WO_3_: 20 0.8407 meq g^−1^	55.8 × 10^−7^ cm^2^ min^−1^	CE: 93%, CR: 62%, EE: 75% (50 mA cm^−2^)	[70]
	Nafion 212	wt% of WO_3_: 0 0.9200 meq g^−1^	13.2 × 10^−7^ cm^2^ min^−1^	CE: 88%, CR: 42%, EE: 65% (50 mA cm^−2^)	
	SPEEK/(WO_3_)_0.20_	wt% of WO_3_: 12.20 1.52 meq g^−1^	1.9 × 10^−7^ cm^2^ min^−1^	CE: 96.4%, CR: 72.5%, EE: 77.5% (30 cycles, 50 mA cm^−2^)	[71]

NA—Not available, * IEC: Ion-exchange capacity, ** PSSS: Poly(sodium 4-styrenesulfonat).

### 3.2. Modified Membranes for Zn-Air RFBs

One of the main challenges in Zn-based FBs is the crossover of active species, mainly zincate ions from the cathode to the anode side, resulting in capacity fade [72,73,74]. Mainly, dense AEM [75] and modified porous membranes [18,19] have been employed to address this issue. The latter strategy, which falls under the scope of the current review work, is discussed below.

**Figure 9:**
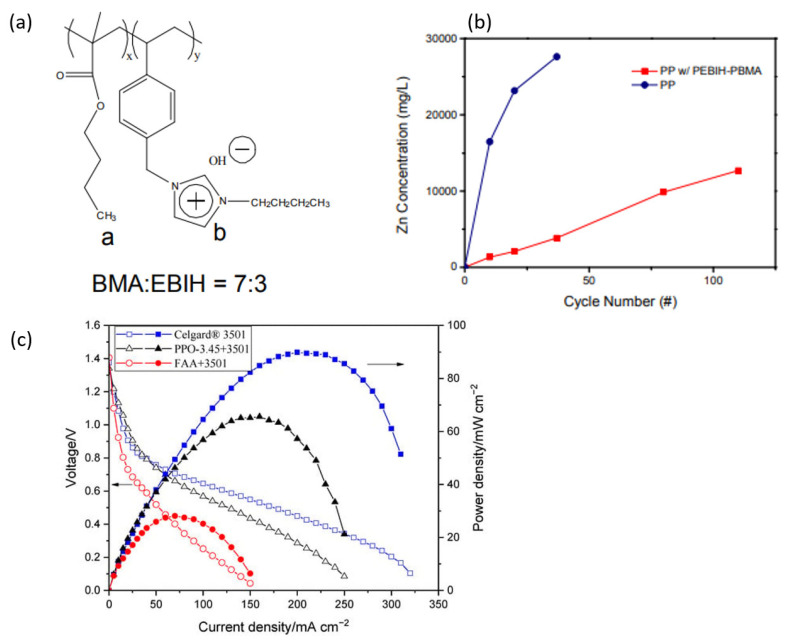
Modified Celgard membranes employed in Zn-based batteries: BMA:EBIH polymer (**a**), (**b**) Zincate ions crossover through Celgard5550 and modified Celgard 5550, and power density of Celgard3501 and modified Celgard 3501-based Zn-slurry flow batteries (**c**). Reproduced with permission from [18,19]. Copyright 2016 American Chemical Society.

The cycling stability of secondary Zn-air batteries was increased when Celgard5550-coated with polymerized ionic liquid was used [19]. The coating was prepared by copolymerizing 1-[(4-ethenylphenyl)methyl]-3-butyl-imidazolium hydroxide (EBIH) and butyl methacrylate (BMA) monomers, as shown in Figure 9a. The zincate ions crossover through the pristine and modified membrane is shown in Figure 9b. The Celgard5550-coated membrane-based cell exhibited much higher charge–discharge cycling (107 vs. 37) with a similar initial energy efficiency. A summary of the discharge and charge test results is provided in Table 4.

Similarly, aiming at reducing the zincate ions crossover in Zn slurry-air RFB, Celgard3501 was coated with two different anion-exchange ionomers in our previous work [18]. The modified membranes were named PPO-3.45+3501 (ionomer based on PPO and spirocyclic quaternary ammonium) and FAA+3501 (based on an ionomer from Fumatech, Fumion FAA3-ionomer). In the PPO-3.45+3501 membrane, the support membrane was coated and impregnated with the polymers, while in the case of FAA+3501, the FAA polymer was completely impregnated into the porous structure of the membrane. The PPO 3.45+3501 membrane had a significantly lower crossover of Zn(OH)_4_^2−^ ions compared to the pristine Celgard^®^ 3501 membrane (5.2 × 10^−13^ vs. 9.2 × 10^−12^ m^2^ s^−1^). The modified membrane-based battery had a high maximum power density of 66 mW cm^−2^, but this was lower than the Celgard^®^ 3501-based cell’s power density of 90 mW cm^−2^ (Figure 9c), likely due to the partial filling of pores with ionomers causing an increase in membrane resistance. However, the modified membranes were not tested in rechargeable batteries.

Overall, these modified membranes show potential for use in rechargeable Zn–air RFBs, but further optimization is needed.

### 3.3. Modified Membranes for AORFBs

**Figure 10:**
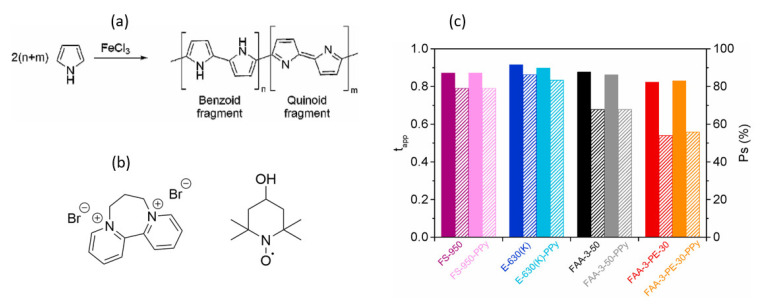
Oxidative polymerization of pyrrole (**a**), chemical structures of BP7 and TEMPOL (**b**), and transport number (filled columns) and permselectivity (dashed columns) of membranes (**c**). Graphs are taken from [77] with permission. Copyright 2021 the authors.

Sanchez et al. [77] modified four commercial ion-exchange membranes (FAA-3-50, FAA-3-PE-30, FS-950 and E−630(K)) with PPy and compared their physicochemical prioerties, ion transport properties and electrochemical properties before and after the membrane modification. The modification was performed via oxidative in situ polymerization of pyrrol monomer using FeCl_3_ as an initiator. The polymerization was achieved by immersing the membranes in a 1:1 solution of pyrrole (0.1 M) and H_2_SO_4_ (0.1 M) for 6 min (to impregnate the membranes with the monomer) followed by immersion in an oxidant-containing solution (FeCl_3_·6H_2_O, 0.5 M) for 18 min (Figure 10a). The crossover of the redox active species through the pristine and modified membranes was determined using viologen derivative (BP7) and hydroxy-2,2,6,6-tetramethylpiperidin-1-oxyl) (TEMPOL) molecules. The permeation of BP7 and TEMPOL through the membranes was reduced by an order of magnitude, despite no significant change in the membrane’s initial ionic conductivity (Table 5). It should be noted that the BP7 molecule had two positive charges, whereas TEMPOL was neutral under the experimental conditions. The chemical structures of the two molecules are shown in Figure 10b.

The permselectivity of membranes was also determined. However, it was demonstrated that the membranes’ transport number and permselectivity before and after PPy modification had a negligible influence on ion-exchange capacity and thus ion selectivity. Even though the modified membranes were promising, they were not tested in AORFB. As a result, the durability and performance are unknown.

Recently, membranes based on polymers of intrinsic microporosity (PIM) with different selective layer thicknesses (0.3–12 µm) were prepared and tested in 2,6-DPPAQ/K_4_Fe(CN)_6_-based aqueous RFBs [78]. The ion selective membrane with a selective layer of 4 µm (named PIM-EA-TB-4.0) exhibited limited water and electrolyte permeation. This led to an RFB with very low capacity fade (about 0.005%/cycle) over 4500 cycles (equivalent to two weeks of charge–discharge cycles). Under the same testing condition, the Nafion 212-based cell demonstrated a cycling performance of 0.017%/cycle. At 80 mA cm^−2^, PIM-EA-TB-4.0-based RFB achieved EE of roughly 65%, just under Nafion 212’s (around 72%).

## 4. Conclusions and Future Perspectives

To sum up, redox flow batteries (RFBs) are highly promising devices that offer many advantages over conventional batteries. Their ability to be upscaled, long lifespan, safety, and eco-friendliness make them suitable for diverse applications, ranging from renewable energy systems and grid-scale energy storage to residential energy storage. With the increasing need for sustainable energy storage solutions, it is expected that RFBs will be increasingly adopted in the future.

In most cases, the fabrication of modified membranes seems to focus on developing membranes with reduced active species crossover via coating/impregnation of polymeric ionomer or inorganic particles on a porous support membrane. So far, modified membranes have been tested in VRFBs and Zn-air RFBs. However, similar membranes can also be used in other RFB chemistries to reduce the crossover of redox active species. Mainly, in AORFB, where mostly organic species are used as reactive chemicals, modified membrane can better block the crossover of such molecules. Similarly, a long-term stability can be achieved with the use of modified membranes in RFBs, such as zinc-polyiodide flow batteries.

Almost all modifications have resulted in reduced active species crossover, thus reducing the capacity fade. On the other hand, an increase in electrical resistance has been observed. However, it must be noted that a specific membrane is required for a defined application or depending on the customer/application requirement. Therefore, such membranes with reduced active species crossover prepared at the cost of slightly/significantly higher resistance might be suitable in some specific cases.

Despite the encouraging results obtained with modified membranes, there are still some remaining challenges. For instance, when a composite membrane is fabricated by the formation of a thin layer of inorganic material on top of a polymeric membrane, delamination can occur, especially when there is a mismatch in the solvent swelling ratio between the two parts. The addition of a compatibilizing additive can enhance the bonding between polymer and inorganic layers. Polydopamine is an example of such an agent that has been employed to improve the adhesion between tungsten oxide and Nafion N117 [79].

It has to be noted that the ultimate objective of using modified membranes is to reduce active species crossover while maintaining a reasonable ionic conductivity in order to improve the cyclability and lifespan of RFBs.

Further research and optimization efforts on the porous substrate membrane, ion-exchange polymer or inorganic particles, and modification strategies are required to advance the current understanding and usage of modified membranes in various RFBs. Similarly, various porous membranes could be tested as base membranes.

Another important issue that is frequently overlooked is the need for testing of the modified membranes in RFBs for their operational stability, cyclability and durability to determine the long-term performance of the coated/impregnated materials.

Last but not least, alternative modification techniques should also be explored, including those that have proved to be successful in other fields of study, such as water treatment. These include layer-by-layer and interfacial polymerization.

## Figures and Tables

**Table 4:** Zincate ion permeability and Zn-air based batteries performance using modified membranes.

Modification Type	Membrane	Property	Application	Zincate Ions Diffusion Coefficient (m^2^ s^−1^)	Battery Performance	Ref.
Pore filling/impregnation	Celgard3501	25 µm-thick Pore size: 64 nm	ZAFB	9.2 × 10^−12^	Cell resistance: 2 Ω cm^2^ Peak power density: 90 mW cm^−2^	[18]
	Celgard3501 + FAA	2 mg cm^−2^ ionomer coating		3.3 × 10^−14^	Cell resistance: 5.6 Ω cm^2^ Peak power density: 28 mW cm^−2^	
	Celgard3501+ PPO-3.45	2 µm-thin layer		5.2 × 10^−13^	Cell resistance: 2.6 Ω cm^2^ Peak power density: 66 mW cm^−2^.	
Pore filling/impregnation	Celgard 5550	25 µm-thick Pore size: 64 nm	Secondary Zn–air battery	5 × 10^−7^	CE = 99.8% Initial EE = 59.4% Cycle = 37	[19]
	PEBIH-PBMA-coated PP separator	25 µm		1.1 × 10^−5^	CE = 99.9% Initial EE = 60.8% Cycle = 107	
Coating with Mn(OH)_2_	Two Celgard^®^ 3401 membranes			6.9 × 10^−12^	NA	[76]
	Two Celgard^®^ 3401 coated with Mn(OH)_2_			6.0 × 10^−15^		

**Table 5:** BP7 and TEMPOL permeabilities through modified PPy-based modified AEMs and CEMs [77]. All membranes were evaluated in NaCl 1 M and tested at room temperature *. Battery test not available.

Membrane	Property	Ion Permeability	
		BP7 Permeability (× 10^10^ cm^2^ s^−1^)	TEMPOL Permeability (× 10^10^ cm^2^ s^−1^)
FS-950	CEM 52 μm-thick 15.8% water uptake 2.2 mS cm^−1^ ion conductivity 1.5 mmol g^−1^ IEC	2.49	96.0
FS-950-PPy	3.2% water uptake 5.3 mS cm^−1^ ion conductivity 0.4 mmol g^−1^ IEC	2.49	30.6
E-630(K)	CEM 34 μm-thick 19.6% water uptake 2.6 mS cm^−1^ ion conductivity 1.1 mmol g^−1^ IEC	122.0	1.21
E-630(K)-PPy	6.1% water uptake 3.5 mS cm^−1^ ion conductivity 0.9 mmol g^−1^ IEC	1.45	0.97
FAA-3-50	AEM 45 μm-thick 15.6% water uptake 1.1 mS cm^−1^ ion conductivity 1.9 mmol g^−1^ IEC	1.26	192.0
FAA-3-50-PPy	6.6% water uptake 3.8 mS cm^−1^ ion conductivity 2.3 mmol g^−1^ IEC	1.26	0.63
FAA-3-PE-30	AEM 23 μm-thick 17.1% water uptake 0.3 mS cm^−1^ ion conductivity 1.1 mmol g^−1^ IEC	5.41	68.8
FAA-3-PE-30-PPy	0.8% water uptake 1.5 mS cm^−1^ ion conductivity 2.0 mmol g^−1^ IEC	1.39	0.62

* Conductivity measurements were performed in a two-chambers cell set-up under flowing wet air at 30 °C.
